# Water Use by Chinese Pine Is Less Conservative but More Closely Regulated Than in Mongolian Scots Pine in a Plantation Forest, on Sandy Soil, in a Semi-Arid Climate

**DOI:** 10.3389/fpls.2021.635022

**Published:** 2021-04-09

**Authors:** Hongzhong Dang, Xueli Zhang, Hui Han, Shuai Chen, Mingyang Li

**Affiliations:** ^1^Institute of Desertification Studies, Chinese Academy of Forestry, Beijing, China; ^2^Institute of Sandy Land Management and Utilization, Shenyang, China

**Keywords:** *Pinus sylvestris* var. *mongolica*, *Pinus tabuliformis*, sap flux density, canopy transpiration, canopy stomatal conductance

## Abstract

The diversity of plant water use patterns among species and ecosystems is a matter of widespread debate. In this study, Chinese pine (*Pinus tabuliformis*, *CP*) and Mongolian Scots pine (*Pinus sylvestris* var. *mongolica*, *MP*), which is co-exist in the shelterbelt plantations in the Horqin Sandyland in northern China, were chosen for comparison of water use traits by monitoring xylem sap flow alongside recordings of the associated environmental factors over four growing seasons. Continuous sap flux density measurements were converted into crown projected area transpiration intensity (T_r_) and canopy stomatal conductance (G_s_). The results indicated that *MP* showed a higher canopy transpiration intensity than in *CP*, with T_r_ daily means (±standard deviation) of 0.84 ± 0.36 and 0.79 ± 0.43 mm⋅d^–1^, respectively (*p* = 0.07). However, the inter-annual variability of daily T_r_ in *MP* was not significant, varying only approximately a 1.1-fold (*p* = 0.29), while inter-annual variation was significant for *CP*, with 1.24-fold variation (*p* < 0.01). In particular, the daily mean T_r_ value for *CP* was approximately 1.7-times higher than that of *MP* under favorable soil moisture conditions, with values for relative extractable soil water within the 0–1.0 m soil layer (REW) being above 0.4. However, as the soil dried out, the value of T_r_ for *CP* decreased more sharply, falling to only approximately 0.5-times the value for *MP* when REW fell to < 0.2. The stronger sensitivity of T_r_ and/or G_s_ to REW, together with the more sensitive response of G_s_ to VPD in *CP*, confirms that *CP* exhibits less conservation of soil water utilization but features a stronger ability to regulate water use. Compared with *MP*, *CP* can better adapt to the dry conditions associated with climate change.

## Introduction

The impact of climate change on both natural and plantation forests has been a concern for some time and has been reported on across the world (Allen et al., 2010; Cook et al., 2018). In particular, concern has been expressed for forests in water-limited areas and in areas where the soil habitats are especially fragile (Klein, 2015). Many factors relating to climate change - more extreme temperatures, increases in pests and diseases, increased numbers and severity of fire events - are causing or at least exacerbating the declines of many forests through increased tree mortality (Martinez-Vilalta and Pinol, 2002; McDowell et al., 2008, 2013; Allen et al., 2010; Giuggiola et al., 2010; Choat et al., 2018). Natural forests are generally more robust than plantation forests. The former are usually better able to resist change due to the complex interactions among the various components of their ecosystems and environments and thus maintain greater stability. Conversely, plantation forests are much simpler ecosystems, and these forests suffer heightened risk of degradation even under relatively minor changes in the interactions of the ecosystem components in their environment. This is especially true in relation to potentially overwhelming problems associated with changes in water availability (Licata et al., 2008; Payn et al., 2015).

Transpiration and canopy stomatal conductance of trees are essential for the quantitative evaluation of severity of drought stress, the magnitude of drought resistance, and the strength of drought resilience of trees (Verbeeck et al., 2007; Heres et al., 2014; Peters et al., 2015; Børja et al., 2016; Macinnis-Ng et al., 2016). In general, canopy stomatal conductance is sensitive to changes in the atmospheric and soil environments (Granier et al., 1999; Nadezhdina, 1999; Bovard et al., 2005; Hernandez-Santana et al., 2016). Trees seem to benefit from decline in canopy stomatal conductance, because sensitive stomatal regulation is critical to prevent trees from approaching the threshold of critical vulnerability to the excessive loss of water (Jones, 1984; Addington et al., 2004). However, the long-term closure of stomata may also bring about carbon starvation (McDowell et al., 2008), resulting in the risk of chronic tree death. Nevertheless, the change in canopy stomatal conductance is broadly used to evaluate the drought-resistance ability of trees, as well as to discover the underlying mechanisms (Poyatos et al., 2013; Meir et al., 2015), such as isohydraulic behavior (McDowell et al., 2008). However, eco-physiological responses to drought show great species-specific variability (Tatarinov et al., 2016; Urban et al., 2019), indicating the diversity of tree hydraulic traits.

In general, *Pinus* is a light-demanding pioneer species and is thus not highly specialized with respect to site conditions (Weber et al., 2007). *Pinus* thus has evolved to avoid competition from other tree species by being able to control transpiration and grow in extreme conditions (Urban et al., 2019). However, *Pinus* trees have been found to be more prone to drought-induced xylem embolism than other conifers (Martinez-Vilalta and Pinol, 2002). Scots pine, as a widely distributed *Pinus* species in Europe (Martinez-Vilalta and Pinol, 2002; Giuggiola et al., 2010), has encountered serious declines and/or tree mortality in many regions (Irvine et al., 1998; Guada et al., 2016). As a result, water use by Scots pine has been observed to be reduced by as much as 60% in 39-year-old Scots pine forests (Llorens et al., 2010) and by up to 65% in 41-year-old Scots pine forests (Irvine et al., 1998).

Northern China is experiencing increases in temperature that are to two or three-times greater than those elsewhere in China, or in the world at large (Stocker et al., 2013). To combat desertification and control dust storms, the Chinese government has implemented a number of large-scale projects, such as the “Three North Shelter Forest Program” and the “Grain for Green Project,” the two of which are also called the “Great Green Wall” projects (Zhu and Zheng, 2019). These projects have directly increased vegetation cover in northern China and contributed to the greening of the world (Chen et al., 2019), and have therefore greatly reduced damage from soil erosion and desertification (Bryan et al., 2018), while their ecological contributions have sometimes been downplayed or doubted (Wang et al., 2010; Zastrow, 2019). However, with rapid and widespread “greening” of the land in China, a series of attendant problems have also arisen, including declines and tree mortality of forests. These issues have now become a popular subject of global discussion (Xu, 2011; Bryan et al., 2018). It is anticipated that the severity of these challenges will only increase under the projected scenario of continuing global climate change (Cook et al., 2018).

Chinese pine (*Pinus tabuliformis, CP*) is a pine species endemic to China, and Mongolian Scots pine (*Pinus sylvestris* var. *mongolica*, *MP*) is one of the geographical varieties of Scots pine; these two *Pinus* species have together served as the lead actors in the ongoing “Great Green Wall” projects, specifically in Horqin Sandyland regions where the shelterbelt afforestation to combat desertification began in 1955 (Liu et al., 2019; Zhu and Zheng, 2019). *MP*, as a pine species introduced to low latitudes to the Horqin Sandyland, exhibits more favorable afforestation survival, growth, and cold tolerance than does the Chinese endemic *CP* pine species (Jiao, 2001; Liu et al., 2019). However, serious declines in the *MP* plantations have been found in the Horqin Sandyland in northern China since 1991 (Jiao, 2001; Zhu et al., 2005), whereas co-existing *CP* plantations are growing healthily. This has stimulated extensive discussions aimed at determining the causes, mechanisms and degradation processes involved in *MP* declines, particularly with respect to tree-water relationships (Zheng et al., 2012; Song et al., 2014; Sun and Liu, 2014; Cai et al., 2020). To date, however, it is not clear how *CP* and *MP* differ in their responses to drought conditions.

The hydraulic diversity of the plants in a plantation ecosystem is important for improving stability by strengthening ecosystem resilience during drought (Anderegg et al., 2018). In northern China, both *CP* and *MP* have been successfully established on sandy soils to control desertification. However, the mechanism underlying the widely reported decline in *MP* as opposed to *CP* and whether a decline in *CP* in the future under the scenario of a changing climate is foreseeable are not clear. We attempt to gain deeper insights into this topic by focusing on the differences in water-use patterns of the two co-existing species. Therefore, the main objectives of this study are: (1) to evaluate annual water use intensity by quantitatively monitoring transpiration of two tree species in multiple years, (2) to compare the differences in response of water use to a range of environmental factors and their gradients at multiple time scales between two co-existing pine species, and (3) to identify water use strategies under drought and differences between the two pine species.

## Materials and Methods

### Study Site

The experiment was conducted at the Zhanggutai National Desertification Control Experimental Station located at the southern edge of the Horqin Sandyland area, Liaoning Province, China (122°22′E, 42°43′N and 226.5 m a.s.l.) during 2013-2018. The climate is semi-arid and continental monsoon. Over the last 30 years, the mean annual air temperature was 7.9°C, the mean frost-free period was 155 days, the mean annual pan evaporation was 1553 mm, and the mean annual precipitation was 475 mm. Approximately, 92% of this precipitation fell from May to September (the main growing season). The soil had an aeolian sand texture, consisting of 84% sand particles (> 0.05 mm), 9% silt particles (0.05–0.002 mm) and 7% clay particles (<0.002 mm). The soil bulk density was 1.61 g⋅cm^–3^, the capillary porosity was 33% and the soil was barren with a mean organic matter content of 0.65 g⋅kg^–1^ in the upper 1.0 m soil layer (Dang et al., 2019a).

### Materials

We selected two types of monocultural plantation forests that both served as shelterbelts, *MP* and *CP*, as sample forests in this study. The two species were planted in the same year, both reaching approximately 46 years of age in 2013. The stand densities were approximately 400−450 stems⋅ha^–1^.

*CP* is a unique coniferous species in China (Jiang et al., 2002; Cai et al., 2020). Natural *CP* forests represent an important forest type in the warm/temperate deciduous forest regions of China (approximately 31°−43°N, 103°20′−124°45′E) (Cai et al., 2020). In this species’ area of natural distribution, the multiple-year annual average temperature is 1−2°C, the recorded highest annual average temperature is 14°C, the average lowest temperature (January) ranges between −20°C and -4°C, the annual precipitation is between 400 and 800 mm, and the altitude ranges from 400 to 1000 m a.s.l. The soil types are mainly cinnamon soil, brown soil and gray-cinnamon soil. The diameter growth rate of natural *CP* trees in forests shows a significant negative correlation with latitude over its entire distribution area and significant positive correlation with average temperature and precipitation in January but does not exhibit any significant correlations with either longitude or altitude.

*MP* is one of the regional varieties of Scots pine, found naturally in Russia, Mongolia and the Chinese Da Xiggan Ling Mountains and Hulunbuir Sand (approximately 47°35′–53°33′N, 118°58′–127°10′E) (Zhao and Li, 1963). The annual average temperature in this species’ natural distribution area is −2.5 to −2°C, the average lowest temperature (January) is −30 to −24°C, the average annual precipitation is between 325-600 mm and the altitude is between 600−400 m a.s.l. The soil types are mainly gray soil and sandy soil. The *MP* trees in natural forests are long-lived and reach maturity at approximately 80-90 years (Zhao and Li, 1963).

### Experimental Layout

Two experimental plots, each enclosed by a 15 m × 15 m fence, were each established in the middle of approximately 30 ha sample forest, one of *CP* and one of *MP*. According to the recommendations for the numbers of sample trees for sap flow measurements (Köstner et al., 1996; Kume et al., 2010), we selected eight sample trees with similar sizes in each plot. The diameter at breast height (DBH) averaged approximately 24.1 ± 2.31 cm (mean ± SE) in the *MP* plot, which was significantly higher than the mean DBH value of 20.8 ± 1.3 cm in the *CP* plot (*p* < 0.01). More detailed information on the sample trees can be seen in Table 1. Measurements for the meteorological variables and soil moistures had continued since 2013, but the sap flow measurements were performed only during the growing season in 4 years, i.e., 2014, 2015, 2017, and 2018.

**TABLE 1:** Diameter at breast height (DBH), tree height (*H*), crown width east-west (BC_E–W_), and south-north (BC_S–N_) for all sap flow-measured trees in sampled in 2013.

**Sample No.**	***Pinus sylvestris* var. *mongolica* (*MP*)**			***P*. *tabuliformis* (*CP*)**		
	**DBH (cm)**	***H* (cm)**	**BC_*E–W*_ × BC_*S–N*_ (m)**	**DBH (cm)**	***H* (cm)**	**BC_*E–W*_ × BC_*S–N*_ (m)**
1	20.02	11.3	4.0 × 3.4	19.1	10.7	4.2 × 4.5
2	22.46	12.7	4.2 × 5.4	19.5	11.0	5.2 × 3.8
3	23.32	12.7	5.3 × 4.9	20.2	7.8	4.0 × 3.9
4	24.02	12.7	5.5 × 6.2	20.6	9.2	4.0 × 4.5
5	24.04	13.2	5.8 × 6.0	20.7	9.7	4.2 × 4.0
6	25.28	12.2	6.8 × 5.0	21.4	10.2	4.8 × 3.8
7	25.80	12.0	6.3 × 5.8	21.6	10.5	5.2 × 5.8
8	27.72	13.2	6.2 × 7.1	23.1	9.4	4.8 × 4.9
Mean	24.08	12.50	5.51 × 5.48	20.78	9.81	4.55 × 4.40
SE	2.31	0.64	0.99 × 1.10	1.27	1.03	0.51 × 0.69

Crown widths were measured east-to-west and south-to-north and are listed in Table 1. These were used to calculate the crown projected areas of the sample trees by applying the following relationships based on to our stand investigation:

For *MP:*

$$A_{c}=0.02865\,D\,B\,H^{2}+0.13872\,D\,B\,H$$

(1) $$+3.38586\left(R^{2}=0.86,n=53\right)$$

For *CP:*

$$A_{c}=0.03132\,D\,B\,H^{2}+0.03345\,D\,B\,H$$

(2) $$+4.32863\left(R^{2}=0.85,n=67\right)$$

where, A_*c*_ (cm^2^) is the crown projected area and DBH (cm) is the DBH (∼1.3 m).

### Meteorological Data

Micrometeorological factors, including radiation, air temperature (T_a_), relative humidity (RH), wind speed and precipitation, were measured by an automatic weather station (AR5, Avalon Scientific, Inc., NJ, United States), located in an open area approximately 50 m away from the experimental plots. Variables were recorded every 10 min using a data logger and subsequently averaged (or summed) to generate hourly and daily values. Hourly vapor pressure deficit (VPD, kPa) was calculated based on T_a_ and RH (Campbell and Norman, 1998).

(3) $$V\,P\,D=0.611\,e^{\left(\frac{17.502\,T_{a}}{T_{a}+240.97}\right)}\,\left(1-R\,H\right)$$

We adopted the standardized precipitation evapotranspiration index (SPEI) to describe the atmospheric drought severity (Vicente-Serrano et al., 2010; Beguería et al., 2014). We calculated the SPEI in the R package SPEI^1^ based on precipitation and temperature data over a 12-month time scale during 2004-2018. We used SPEI to categorize dry and wet gradings according to the standards (Chen and Sun, 2015).

### Soil Moisture Measurements

We measured the volumetric soil water content (θ, cm^3^⋅cm^–3^) at 20-cm intervals in the upper 1.0 m soil layer at three locations in each experimental plot with ECH_2_O EC-5 probes (METER Group, Inc., Pullman, WA, United States). The data were collected at 10 min intervals and averaged to hourly or daily scales. The sensor readings were site-specific and were calibrated using the following formula based on the soil-core method: θ = 0.99421 × θ_*sensor*_ + 0.00128 (*R*^2^_*adj*_ = 0.94, *n* = 202, *p* < 0.001). We calculated the relative extractable soil water (REW), which is defined as the quotient of the actual extractable water to the maximum extractable water, to describe the relative soil moisture conditions at the site (Granier et al., 1999):

(4) $$R\,E\,W=\frac{\bar{\theta }-\theta_{\min}}{\theta_{f\,c}-\theta_{\min}}$$

where, θ_*fc*_ (%) is the field capacity (17.5% in the 0.0–1.0 m soil depth based on field observations) and θ_*min*_ is the minimum soil moisture during the experimental period (2.3%). $\bar{\theta }$ is the mean of soil moisture values from the corresponding soil layers (%). We adopted the threshold value of REW = 0.4 recommended in several reports to define soil water stress (Granier et al., 1999; Bernier et al., 2002; Poyatos et al., 2005), which corresponds to the θ value of 0.086 cm^3^⋅cm^–3^ at our site. A more detailed threshold value of REW was later deduced from the model based on our field measurements.

### Sap Flux Density Measurements and Canopy Transpiration Estimation

Sap flux density (J_s_, cm⋅s^–1^) in the outer 3 cm width xylem layer was measured continuously using thermal dissipation sensors (Dynamax Inc., Houston. TX. United States). We installed the sensors 1.3 m above the soil level on the north sides of the stems. The distance between the two probes of each sensor was 0.04 m (see the specifications in the brochure)^2^. The upper needle of a probe was heated with a constant power of 0.2 W. The sensors were shielded with reflective foil that extended 1.0 m below and 0.5 m above to minimize effects from incident radiation. We sealed the foil with the stem above the installation to prevent ingress of raindrops and stem-flow water. The temperature difference between the two probes was measured at 10-min intervals and recorded every hour using SQ2040 data loggers (Grant Instruments Ltd, Cambridge, United Kingdom). The measurements were recorded during an entire growing season each year. At the end of the growing season, we removed all probes from the trees and reinstalled them at the beginning of the next growing season (in early April) to minimize possible signal dampening (Moore et al., 2010). J_s_ was calculated using Granier’s original equation (Granier, 1987):

(5) $$J_{s}=119\times 10^{-4}\,{\left(\frac{\Delta \,T_{0}-\Delta \,T}{\Delta \,T}\right)}^{1.231}$$

where, Δ*T* is the measured temperature difference between the heated and reference needles. Δ*T*_0_ is the maximum Δ*T* when the sap flux density is close to zero, which is determined over approximately 10 consecutive measuring days, using a linear regression (Lu et al., 2004).

The total sap flow through the section of trunk instrumented was considered to be equal to the total transpiration from the canopy (Köstner et al., 1996). The canopy transpiration intensity (T_*r*_, mm⋅day^–1^) was calculated based on the measured sap flux density (J_*s*_, cm⋅s^–1^), the sapwood area (A_*s*_, cm^2^) at the instrumented section, and the projected area of the crown (A_*c*_, m^2^):

(6) $$T_{r}=\sum_{j=1}^{n}\sum_{i=1}^{24}\left(J_{s,i,j}\times A_{s,j}\times 3600\right)/A_{c,j}/1000/n$$

where, *n* is the number of sample trees (*n* = 8 for each of the two species in the study). J_*s,i,j*_ is the measured sap flux density in the outer 3 cm width of xylem of the *j*th-tree at the *i*th-hour. A_*s,j*_ is the sapwood area of the *j*th-tree calculated by Eq. (7) or Eq. (8) based on the DBH values. A*_*c*_*_,j_ is the projected crown area of the *j*th-tree calculated by Eq. (1) or Eq. (2) based on the DBH values.

For *MP:*

As= 0.7117×DBH1.9472(R=20.99,n=25

(7) (Han et al., 2013)

For *CP:*

$$A_{s}=0.8244\times DBH^{1.9494}\left(R^{2}=0.99,n=28\right)$$

(8) (Ma et al., 2001)

where, A_*s*_ (cm^2^) is the sapwood area and DBH (cm) is the DBH (∼1.3 m).

In 2018, due to the great decrease in battery power capacity, insufficiency of sensor power occurred on some days in two independently observed plots, resulting in effective data being obtained only during the daytime when the sunlight rose to relatively strong levels. Days when these data on any one plot were incomplete were excluded when calculating daily T_*r*_ for comparisons between tree species.

### Canopy Stomatal Conductance

We calculated canopy stomatal conductance (G_*s*_, cm**⋅**s^–1^) from canopy transpiration (T_*r*_) on the crown projected area basis and VPD using the simplification of the inversion of the Penman-Monteith model (Monteith and Unsworth, 1990). G_*s*_ was calculated on an hourly basis:

(9) $$G_{s}=\frac{\lambda \,T_{r}\,\gamma }{\rho \,c_{p}\,V\,P\,D}\times \frac{1}{36}$$

where, *λ* is the latent heat of vaporization of water (MJ**⋅**kg^–1^), *γ* is the psychrometric constant (kPa**⋅**°C^–1^); *ρ* is the density of air (kg**⋅**m^–3^) and *c*_*p*_ is the specific heat of air (MJ**⋅**kg^–1^**⋅**°C^–1^).

### Statistical Analyses

We defined the growing season for these pines at the site as being for the 6-month period from May 1 to October 30. We calculated G_*s*_ only when the daytime VPD was greater than 0.6 kPa (Ewers and Oren, 2000). Daytime was defined as when solar radiation exceeded 50 W**⋅**m^–2^ (May and June). Considering the incompleteness of recordings of sap flow on some days in 2018, the maximum of hourly sap flux density in a day (J_*s–max*_) was use to describe the xylem sap flow capacity, while only the days with complete recordings of sap flow were selected to calculate canopy transpiration. Average values of J_*s*_, T_*r*_, and G_*s*_ for sample trees were compared between tree species at different timescales using repeated one-way ANOVA at *p* < 0.05 or *p* < 0.01 significance level. Relationships between the variables studied were evaluated using correction and simple and nonlinear regression analyses. The daily T_*r*_ values of the two pine trees were linearly fitted monthly, and the slope *k* was derived. The relationships in which the slope *k* declined with the decreasing of REW were fitted with an exponential equation. The relationships between G_*s*_ and VPD were analyzed using boundary-line analysis performed with quantile regression in the statistical R package Quantreg^3^. We adopted Lohammar’s function (Oren et al., 1999) and the 95th quantile to reflect the boundary-line relationship between Gs and VPD. All statistical analyses were conducted with OriginPro (Version 2021, OriginLan Inc., Northampton, MA, United States).

## Results

### Environmental Factors

**FIGURE 1:**
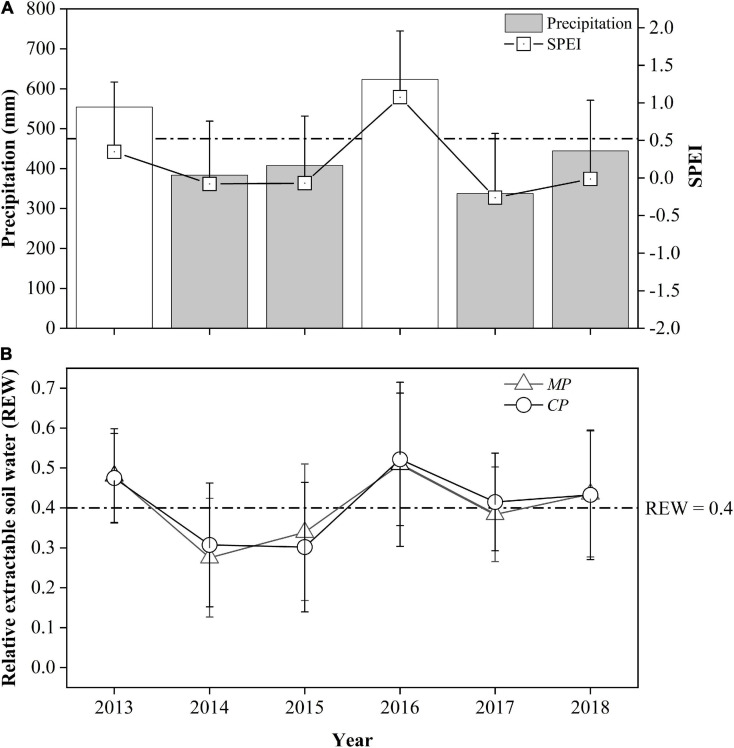
Comparison of annual precipitation, the standardized precipitation evapotranspiration index (SPEI) at the study site **(A)**, and relative extractable soil water in two plots: Chinese pine (*CP*) plot and Mongolian Scots pine (*MP*) plot, **(B)** between years during the study period. The values in 2013 and 2016 are displayed with transparent color where there were no xylem sap flux measurements. The dotted horizontal lines represent the 30-year average precipitation **(A)** and the threshold value of soil water availability at REW = 0.4 **(B)**, respectively.

During the 6-year period from 2013 to 2018, the annual precipitation in 2013 and in 2016 exceeded the 30-year average by approximately 17 and 31%, respectively, but in 2014, 2015, 2017, and 2018, it accounted for 81, 86, 71, and 93% of the 30-year average, respectively. These results indicate 4 years of relative drought from the perspective of annual precipitation. However, more specifically, the drought year sequence seems to have been interrupted by wet years, thus dividing the drought year sequence into two 2-year-long sets. The SPEI values for these 6 years were between −0.26 and 0.35, so their inter-annual variation was essentially consistent with the trend for the annual precipitation (Figure 1A). However, these 6 years belonged to the ‘near normal’ category except for 2016, which belonged to the ‘moderately wet’ category based on the SPEI.

The relative extractable soil water within the 0–1.0 m depth layer (REW) for both sites during the 6-year period varied with annual precipitation, indicating the direct effects of precipitation on soil moisture. In 2014, the REW for the *MP* plot was significantly lower than that for *CP*, 0.28 to 0.31 (*p* = 0.04), but in 2015, the REW in *MP* (0.34) was significantly higher than that in *CP* (0.30) (*p* = 0.03), indicating a small decrease in soil moisture in *CP*, while there was a 12% increase in precipitation during the 2014-2015 period. In 2017, the REW for the *MP* plot averaged 0.38, which was significantly lower than the value of 0.41 for the *CP* plot (*p* = 0.01). In 2018, the REW levels in both the *MP* and *CP* plots increased above 0.43 with a 38% increase in precipitation (Figure 1B). Although the total amount of precipitation in 2017 was the lowest in the 4-year period, the soil moisture at the two sites was better than that during the 2014−2015 period due to the greater precipitation in 2016 (623.6 mm).

**FIGURE 2:**
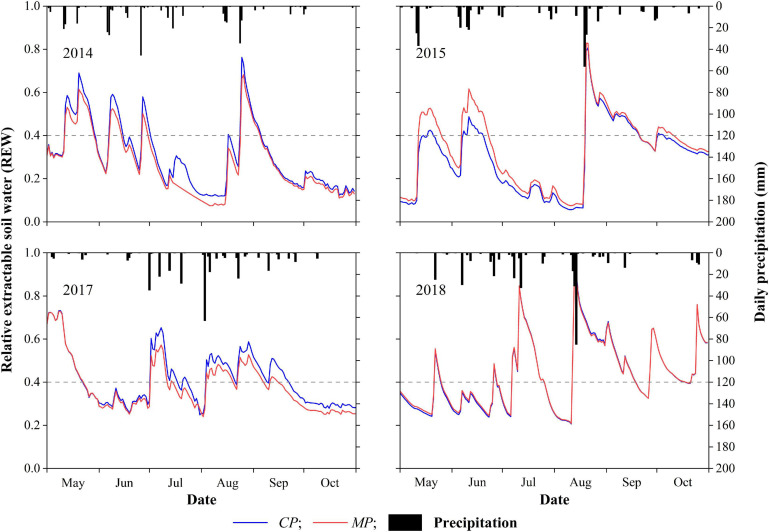
Inter-seasonal dynamics of daily precipitation and daily relative extractable soil water (REW) in the 4 years of xylem sap flux measurement. The dotted horizontal lines represent the threshold value of soil water availability at REW = 0.4.

The inter-seasonal dynamics of daily REW in each year of the 4-year period showed similar patterns at both the *MP* and *CP* sites (Figure 2). Calculations indicate that the drought days for REW < 0.4 at the *CP* site were 138, 132, 96, and 90 days in 2014, 2015, 2017, and 2018, these accounting for 75, 72, 52, and 49%, respectively, of the total days in the 6-month growing season (May 1 to October 31). The drought days for REW < 0.4 in *MP* were 145, 111, 115, and 88 days, in 2014, 2015, 2017, and 2018, accounting for 79, 60, 63, and 48%, respectively, of the total days in the 6-month growing season. Specifically, in 2014 and 2015, the number of days with REW < 0.2 accounted for more than 33% of the growing season at the *CP* site and approximately 30% at the *MP* site. The minimum daily REW during the 4-year period at the *MP* site appeared in 2014 with a value of 0.07, while the daily REW at the same time at the *CP* site was 0.12. The minimum daily average REW during the 4-year period in *CP* was 0.06 in 2015, with the daily average REW in *MP* at that time being 0.08 (Figure 2).

### J_*s–max*_ in a Day

**FIGURE 3:**
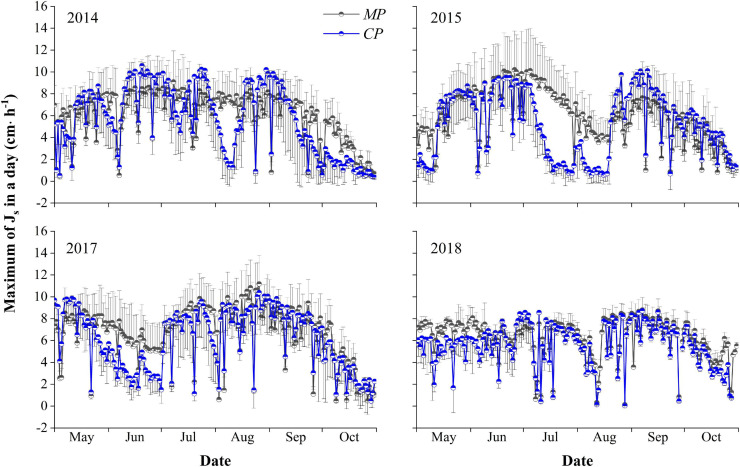
Inter-seasonal dynamics of the maximum hourly sap flux density (J_*s–max*_, cm⋅h**^−^** ^1^) in a day in the 4 years of xylem sap flux measurement for the Mongolian Scots pine (*MP*) and Chinese pine (*CP*) trees. The half circle points represent the means of J_*s–max*_, and short T-type line represent error bars. Only the plus or minus direction error bars are drawn for the clartity of the plot.

The average hourly J_*s–max*_ during the entire growing season over the 4 years averaged 6.21 ± 1.72 cm⋅h^–1^ for *MP*, with a maximum of 11.18 cm⋅h^–1^. The average was significantly higher than the average of 5.60 ± 4.86 cm⋅h^–1^ for *CP* (*p* < 0.05), with a maximum of 10.57 cm⋅h^–1^. There were significant differences in the J_*s–max*_ for *MP* over the 4 years of 2014, 2015, 2017, and 2018, with values of 6.00 ± 2.31, 5.93 ± 2.44, 6.66 ± 2.58, and 6.23 ± 1.76 cm⋅h^–1^, respectively (*p* < 0.05). Similarly, significant differences were found for *CP*, with average hourly J_*s–max*_ values of 5.61 ± 3.15, 5.12 ± 3.04, 6.07 ± 2.76, and 5.61 ± 1.98 cm⋅h^–1^, in 2014, 2015, 2017, and 2018, respectively (*p* < 0.05). The average hourly J_*s–max*_ in trees of both *MP* and *CP* varied between years, with the maximum in 2017 and the minimum in 2015. The average hourly J_*s–max*_ for *MP* each year was higher than that for *CP*. However, the difference was significant (*p* < 0.05) only in 2015 (Figure 3).

### T_*r*_ and G_*s*_

**FIGURE 4:**
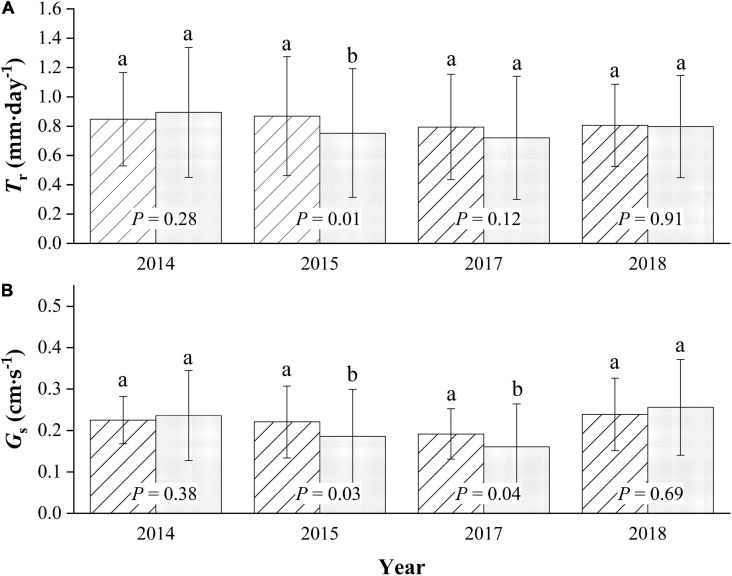
Seasonal comparison of average daily canopy transpiration, T_*r*_ **(A)**, and canopy conductance, G_*s*_ **(B)** for Mongolian Scots pine (*MP*) and Chinese pine (*CP*) in the 4 years of xylem sap flux measurement. Different lowercase letters represented significant differences at α = 0.05 level.

The average daily T_*r*_ of the sampled trees during the entire growing season in the 4-year trial period averaged 0.84 ± 0.36 mm⋅d^–1^, with a maximum of 1.72 mm⋅d^–1^ for *MP*. The average is slightly higher than the average daily T_*r*_ value of 0.79 ± 0.43 mm⋅d^–1^ for *CP* (*p* = 0.07), with a maximum of 1.77 mm⋅d^–1^. There were no significant differences in the daily canopy transpiration between years during the four-season study (*p* = 0.29), with the average daily T_*r*_ values being approximately 0.85 ± 0.32, 0.87 ± 0.41, 0.79 ± 0.36, and 0.81 ± 0.28 mm⋅d^–1^ for *MP*, for the seasons 2014, 2015, 2017, and 2018, respectively. Meanwhile, the differences were very significant for *CP* (*p* < 0.01), with daily average T_*r*_ values of approximately 0.89 ± 0.44, 0.75 ± 0.44, 0.72 ± 0.42, and 0.80 ± 0.35 mm⋅d^–1^ in 2014, 2015, 2017, and 2018, respectively, with a maximum in 2014 and a minimum in 2017 (Figure 4A).

Generally, the canopy stomatal conductance during each growing season in the four-season study in *MP* was slightly higher than that in *CP* (*p* = 0.07), with the average daily G_*s*_ in the two species being approximately 0.22 ± 0.07 and 0.20 ± 0.11 cm⋅s^–1^, respectively. Additionally, there were significant differences in the inter-annual variation in G_*s*_ between years (*p* = 0.01) in *MP*, with daily averages of approximately 0.22 ± 0.06, 0.22 ± 0.09, 0.19 ± 0.06, and 0.24 ± 0.09 cm⋅s^–1^ in the four seasons, respectively. The highest value was in 2018, and the lowest was in 2017. More significant inter-seasonal variation patterns were found in *CP* (*p* < 0.01), with average daily values in G_*s*_ being approximately 0.24 ± 0.11, 0.19 ± 0.11, 0.16 ± 0.10, and 0.26 ± 0.12 cm⋅s^–1^. The average daily value for G_*s*_ in *MP* was significantly higher than that in *CP* only in 2015 and in 2017, while it was slightly lower in 2014 and 2018 (Figure 4B).

### Relationships of T_*r*_ Between Tree Species Varied With REW Levels

**FIGURE 5:**
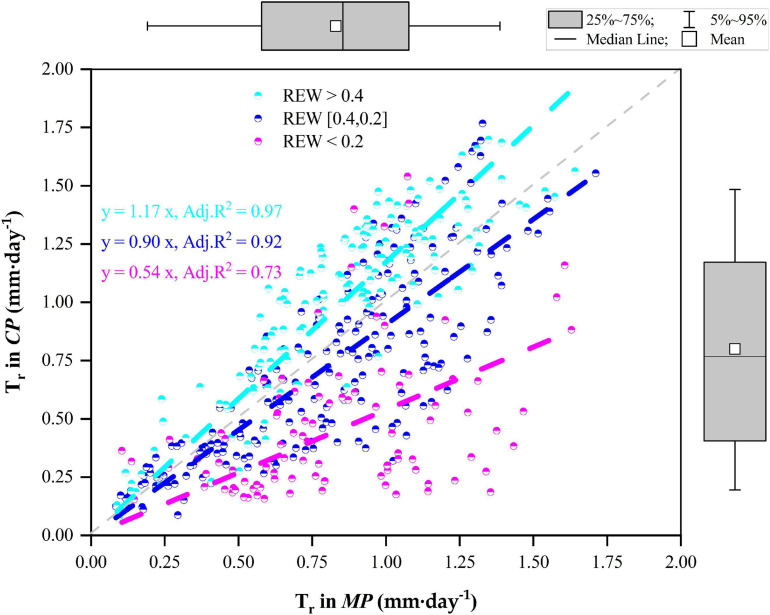
Relationships of canopy transpiration (T_*r*_) between Mongolian Scots pine (*MP*) and Chinese pine (*CP*) in relation to different levels of relative extractable soil water (REW).

The statistics on the daily T_*r*_ of the two pine trees during the whole measurement period as well as their relationships are given in Figure 5. In general, the daily T_*r*_ in *MP* varied positively with the daily T_*r*_ in *CP* during the four growing seasons. When REW > 0.4, the average daily T_*r*_ was approximately 1.03 ± 0.37 mm⋅d^–1^ in *CP*, which was significantly higher than that in *MP* (*p* < 0.01), averaging 0.86 ± 0.33 mm⋅d^–1^. However, when REW was in the range of 0.2–0.4, T_*r*_ in *CP* was lower than that in *MP* (*p* = 0.01), with daily T_*r*_ values of 0.72 ± 0.41 mm⋅d^–1^ compared with 0.81 ± 0.36 mm⋅d^–1^. In particular, when REW < 0.2, the daily T_*r*_ in *CP* was significantly lower than that in *MP* (*p* < 0.01), with values of 0.47 ± 0.31 mm⋅d^–1^ compared with 0.83 ± 0.34 mm⋅d^–1^. The slopes of the linear regressions decreased with decreasing REW levels (Figure 5).

**FIGURE 6:**
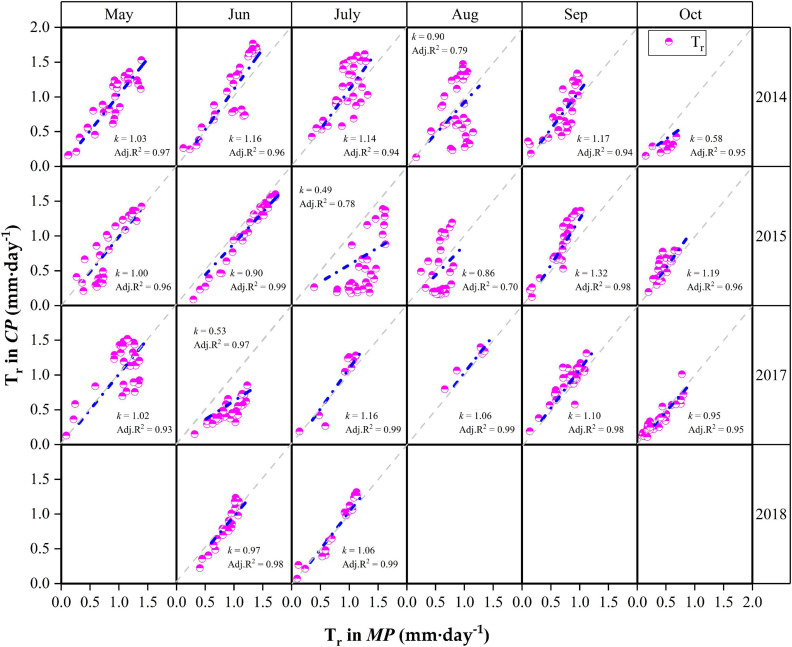
Relationships of canopy transpiration (T_*r*_) between Mongolian Scots pine (*MP*) and Chinese pine (*CP*) in different months during the 4 years of xylem sap flux measurement. *k* is the slope of the linear regression. The gray dotted lines are 1:1 line. Data in May, August and September in 2018 were lost due to faulty cable in *CP* sample trees.

**FIGURE 7:**
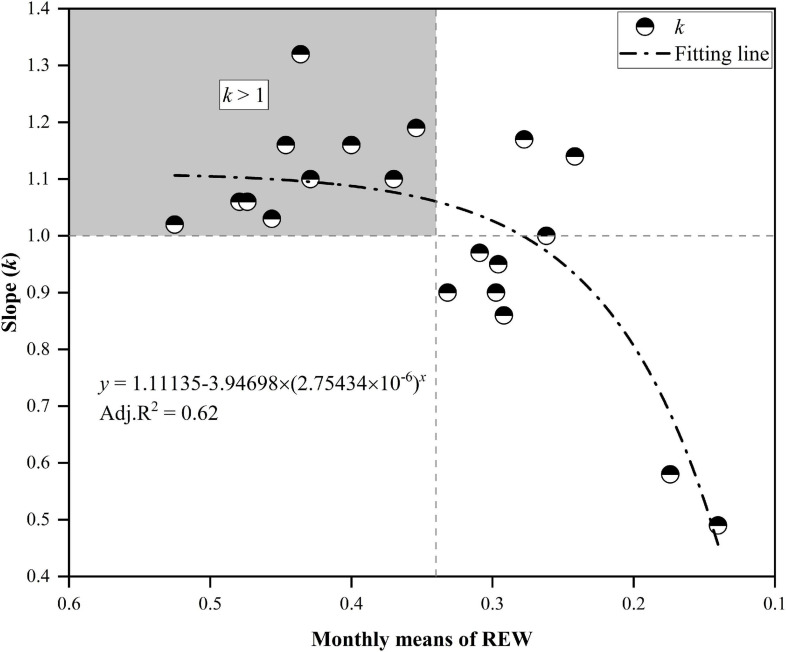
The slope *k* decreased exponentially with the decline in relative extractable soil water (REW). The upper-left block indicates that the canopy transpiration of Chinese pine (*CP*) was generally higher than that of Mongolian Scots pine (*MP*) when REW was higher than approximately 0.34. The canopy transpiration of *CP* was below that of *MP* when REW fell below approximately 0.28.

Detailed descriptions of the relationships for daily T_*r*_ between *MP* and *CP* at the monthly scale are presented in Figure 6. Although the daily T_*r*_ in *CP* was slightly higher than that in *MP* under water-favorable conditions, the opposite was true when soil moisture was lower. For example, the daily T_*r*_ in *CP* was lower than that in *MP* in October 2014 when the REW values at the two sites were low (REW = 0.16–0.17). Similar cases occurred in July 2015 when REW was 0.17 at the *MP* sites and approximately 0.14 at the *CP* sites. Again, in June 2017, the REW at the two sites was approximately 0.30–0.31 (Figure 6). Analyses show that the slope (*k*) of the linear regressions at the monthly scale decreased exponentially with declining REW (Figure 7). The daily T_*r*_ in *CP* was higher than T_*r*_ in *CP* with *k* > 1 given that at REW above 0.34.

### T_*r*_ Diurnal Patterns Changed With Drought

**FIGURE 8:**
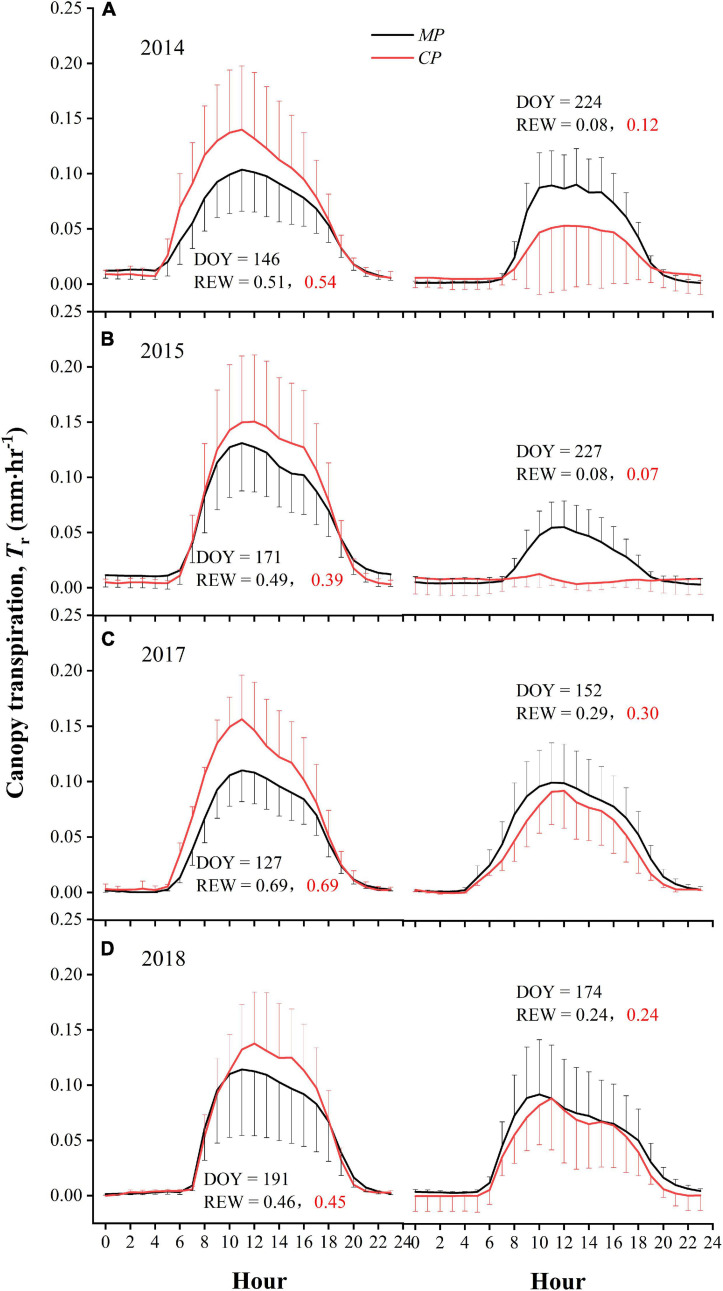
Comparison of diurnal patterns of canopy transpiration, T_*r*_ on two typical sunny days with very different relative extractable soil water (REW) values in 2014 **(A)**, 2015 **(B)**, 2017 **(C)**, and 2018 **(D)** of measurement.

In each growing season, we selected two sunny days with differing but typical levels of soil moisture to explore the possible differences between the two species in diurnal patterns of T_*r*_ in response to drought. On the two selected days in 2014, during which REW had dropped from approximately 0.51–0.54 to 0.08–0.12, the hourly T_*r*_ decreased significantly (*p* < 0.01) in the *CP* plot, with the peak value of T_*r*_ decreasing by 62%, while it decreased only slightly (*p* = 0.16) in the *MP* stand, with the peak value of T_*r*_ declining by only 13% (Figure 8A). On the 2 days in 2015, when REW had declined from a relatively high value of approximately 0.39–0.49 to a very low value of approximately 0.07–0.08, a similar significant reduction in hourly T_*r*_ was found in both *CP* and *MP* trees (*p* < 0.01); the peak T_*r*_ values fell by 92% and 58%, respectively (Figure 8B). However, there were only slight reductions on the 2 days in 2017, when REW dropped from 0.69 to 0.30 in both *MP* (*p* = 0.95) and *CP* (*p* = 0.07) plots. Meanwhile, the peak values of T_*r*_ dropped by 10% and 41%, respectively (Figure 8C). A nearly identical pattern occurred on the 2 days in 2018, when REW dropped from approximately 0.45–0.46 to 0.24, with the hourly T_*r*_ value differing insignificantly for both *MP* (*p* = 0.53) and *CP* (*p* = 0.15) trees, and the peak T_*r*_ values fell by 20% and 36%, respectively (Figure 8D). We conclude that the peak value of T_*r*_ on any day was typically higher in *CP* than in *MP* plot when soil moisture was not under drought status (i.e., REW > 0.4). This was the case in each of the 4 years of our study. Conversely, when soil moisture was under drought (REW ≤ 0.4), the daily peak value of T_*r*_ was lower for *CP* than for *MP*.

### Relationships Between Daily G_*s*_ and REW

**FIGURE 9:**
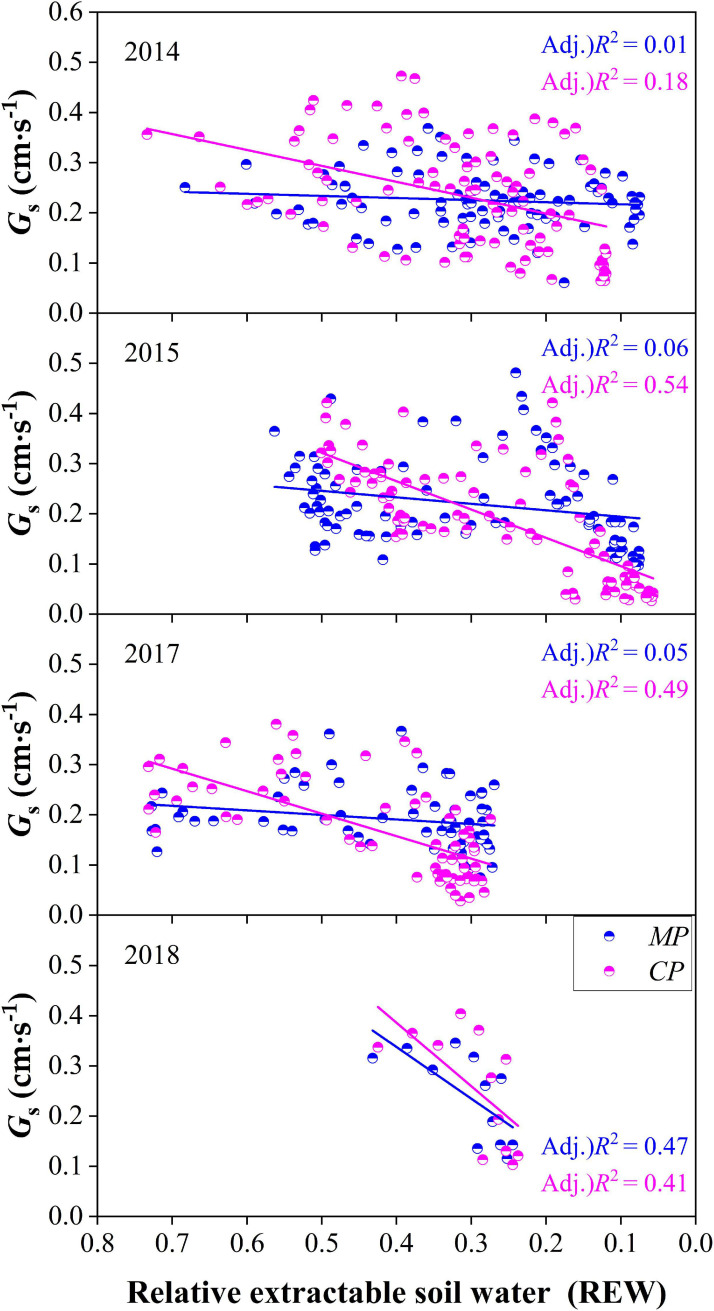
Comparison of canopy conductance, G_*s*_ varied with the level of relative extractable soil water (REW) between Chinese pine (*CP*) and Mongolian Scots pine (*MP*) in different years.

On the daily time scale, G_*s*_ of trees in both *CP* and *MP* generally decreased with the decline in REW each year. However, G_*s*_ responded to the decline of REW more quickly in *CP* than in *MP* in each year according to the slopes of linear regressions at the daily scale, indicating a tighter regulation in response to drought in *CP* than in *MP*, which was usually considered isohydric behavior (Figure 9). The daily G_*s*_ showed a poor relationship with REW in *MP* according to the coefficients of determination from linear regression in each year except for in 2018.

### Relationships Between Hourly G_*s*_ and VPD

**FIGURE 10:**
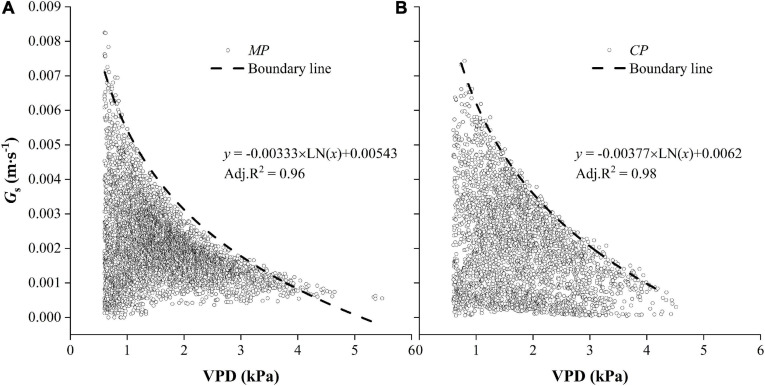
Hourly values of canopy conductance, G_*s*_ varied with vapor pressure deficit, VPD for two tree species Mongolian Scots pine (*MP*) **(A)** and Chinese pine (*CP*) **(B)**. Data were selected when daytime VPD was higher than 0.6 kPa. The dotted line shows the fitted boundary curves suing a logarithmic function.

The hourly G_*s*_ generally decreased with the increasing of VPD in both *CP* and *MP* (Figure 10). In particular, there were clear boundary lines in G_*s*_ that varied with VPD. The boundary was well fitted with a logarithmic function, from which we deduced the parameters and calculated a new variable, G_*s–ref*_, which was defined as the upper value of canopy conductance when the VPD was equal to 1.0 kPa. G_*s–ref*_ was 14.2% higher in *CP* than in *MP*, indicating greater water-consumer behavior in *CP.* Another parameter (*m*), which was used to describe the sensitivity of canopy conductance to VPD, was 13.2% higher in *CP* than in *MP*.

## Discussion

### Water Use Intensity of Individuals

In this study, our results indicated that the water use intensity of *MP* and *CP* individuals in semi-arid sandy land was at relatively low levels, with canopy transpiration averaging 0.84 and 0.79 mm⋅d^–1^, respectively, during the 4-year period based on sap flow measurements. We estimate that these averages amount to transpiration levels of approximately 155 mm and 145 mm transpiration during the entire growing season of each year, which account for approximately 39% and 37% of annual precipitation in the 4 years, respectively. We found that the water use intensity of *MP* was slightly higher than that of *CP* individuals of the same age, while the tree sizes were significantly higher for *MP* than for *CP* individuals due to the more rapid growth of *MP* (*p* < 0.01). We deduce that the overall water use efficiency of *MP* individuals should be higher than that of *CP* individuals in sandy habitat. The faster growth in *MP* in comparison to *CP* at our site is consistent with many previous reports, which have shown that both the aboveground and belowground biomass are generally higher in *MP* than in *CP* of the same age (Zhao and Li, 1963; Zhu et al., 2005). These physiological differences are especially obvious in harsh habitats (Zhu et al., 2005). The fewer fine roots in *CP* help explain why the water use sensitivity to drought in *CP* was higher than that in *MP*. Measurements at the leaf scale show that *MP* features a higher photosynthetic rate, lower transpiration rate, and higher water use efficiency than *CP* under favorable soil moisture conditions (Bai et al., 2008; Ding et al., 2011). However, this trend would change under severely dry soil conditions (Liu et al., 2019). Combining these pieces of information, it can be concluded that the drought stress seems to be more harmful to the needles of *CP*, while it imparts greater damage to the fine roots of *MP*.

### Hydric Behavior in the Two Pine Species

In this study, we compared water consumption of two co-existing pine species by monitoring xylem sap flow and accompanying environmental factors during a 4-year period. In general, both *CP* and *MP* in this semi-arid sandy land showed a conservative water consumption and excellent regulation of canopy stomatal conductance, which basically agrees with findings of previous studies (Song et al., 2014, 2020; Wen et al., 2017; Tang et al., 2018; Wu et al., 2018; Dang et al., 2019a). Our results provide evidence that both *CP* and *MP* exhibit strong drought resistance, like that of Scots pine, compared with other local tree species (Poyatos et al., 2005, 2008, 2013; Macinnis-Ng et al., 2016; Urban et al., 2019). We thus conclude that *CP* is similar to *MP*, and both should be considered water-savers based on their relatively low levels of canopy transpiration. Pines tend to be more homogeneous in their vulnerability to embolism than other conifers (Martinez-Vilalta and Pinol, 2002). The homogeneity of the conducting system of pines may compromise the ability of ecosystems to resist the expected increase in aridity due to climate change.

Isohydric and anisohydric behaviors in species have been widely discussed to describe their water use strategies under water stress (McDowell et al., 2008). In general, isohydric behavior is characterized by decreases in stomatal conductance to maintain nearly constant leaf water potentials (Tardieu and Simonneau, 1998; McDowell et al., 2008; Garcia-Forner et al., 2017). Many species, including Scots pine, have been regarded as isohydric species (Irvine et al., 1998; Llorens et al., 2010; Leo et al., 2013; Urban et al., 2019). Although the concept of fixed hydraulic behavior is being challenged by studies showing that individual plants or species can vary along a continuum spanning isohydric to anisohydric (Ogle et al., 2012; Feng et al., 2019; Guo and Ogle, 2019) and are affected by plant-environment interactions (Hochberg et al., 2018), we think it is a helpful framework concept if the aims are limited to comparisons of water use patterns in co-existing species.

In previous studies, pine species such as Scots pine have been shown to conserve water via sensitive stomatal regulation and to maintain relatively constant shoot water potentials under drying conditions compared with other genera such as *Larix* (Dulamsuren et al., 2009; Urban et al., 2019) and *Quercus* (Poyatos et al., 2008; Chirino et al., 2011; Morán-López et al., 2014). In this study, we conclude that both *CP* and *MP* display typical isohydric behavior by analyzing G_*s*_. Specifically, *MP* individuals have been shown to exhibit a remarkable ability to maintain relatively constant water potentials by reducing stomatal conductance, which is consistent with findings from previous reports (Dang et al., 2019a,b; Song et al., 2020). It has been reported that *CP* maintains its leaf water potential at a relatively constant level of approximately −1.7 MPa by markedly reducing its leaf stomatal conductance (Wen et al., 2017; Tang et al., 2018; Wu et al., 2018). Direct measurements at the leaf scale also indicate that both *CP* and *MP* may be drought-tolerant tree species with high water potential and delayed dehydration (Tang et al., 2018); their leaves exhibit relatively strong water retention capacity and an ability to maintain turgor (Ding et al., 2011; Liu et al., 2019).

### Species-Specific Sensitivity to Dryness

Interspecific variation related to water use patterns was confirmed in our study. Compared with *MP*, we found significantly higher canopy transpiration and canopy conductance values for *CP* individuals when soil water was favorable but sharper reductions under drought conditions. This behavior led to relatively lower annual water consumption by *CP* trees than by *MP* trees in this semi-arid sandy habitat. The results imply that *CP* is more sensitive to soil-water conditions (REW) and atmospheric water conditions (VPD) than is *MP.* These findings provide an explanation for the tighter stomatal regulation in *CP* than in *MP*, enabling the former to cope better with low REW and high VPD and to exhibit stronger adaptation in future projected dry environmental conditions. This interpretation is directly supported by certain case study comparisons of stomatal conductance (Quan et al., 2004) and sap-flow (Yan et al., 2018) between these two species.

In this study, our results indicate that *CP* exhibits a more isohydric behavior than *MP* by making a direct comparison of G_*s*_ from an individual perspective. However, this conclusion does not signify with certainty that the drought resistance of *CP* is stronger than that of *MP*. Previous studies at the level of organs such as needles and/or of seedlings indicate that the indexes such as the ratio of bound water to free water of needles, the water content of needles, the predawn leaf water potential, and the relative permeability of plasmalemma of needles are higher in *MP* than values of these parameters in *CP* (Bai et al., 2008). These results imply that the needles of *MP* feature greater drought-tolerant attributes than those of *CP*, with delayed dehydration to maintain high water potential through biochemical response (Wang et al., 2015). The drought resistance of trees is a complex trait, resulting from morphology, physiological and biochemical response characteristics, cellular photosynthetic organelles, and protoplasm structure, etc. Only by comprehensive studies from different perspectives can we draw more objective conclusions.

### Water Use Strategies Related to Root Distribution

Although the fine control of stomatal openness in isohydric behavior has been considered an important mechanism in drought resistance, isohydric species are often considered to suffer from carbon depletion caused by excessive stomatal closure in the course of multi-annual droughts, resulting in long-lasting growth decline and finally tree death (Buras et al., 2018). Alternatively, trees may depend on other nonstomatal mechanisms, such as water absorption by widely distributed roots, to improve drought resistance in coordination (Konôpka et al., 2005).

The ability of roots to access deep soil water has been found to be crucial to a species’ adaptation to drought (Jiang et al., 2020). Although the roots of larger diameter generally account for a greater proportion of root biomass, approximately 92% of the mineral nutrients and 75% of the water that supports growth is absorbed by fine roots in lateral roots rather than by taproots (Makkonen and Helmisaari, 2001; Epron and Osawa, 2017). Both *MP* and *CP* are shallow-rooted with nearly 85−90% of roots within the upper 60-cm soil layer (Jiang et al., 2002; Xue, 2003). This shallow-rooted feature enhances the ability of trees to use precipitation opportunistically but is not helpful for resisting severe drought because fine roots would die almost immediately when soil turns dry (Vanguelova and Kennedy, 2007), thus directly leading to a reduction in soil-root water transport (Konôpka et al., 2005). This explains why the shallow-rooted trees can easily suffer from drought stress when drought occurs in *MP* forests (Song et al., 2015, 2018) or *CP* forests (Zhou and Shangguan, 2007). In contrast, the water use strategy of *MP* individuals is more prominently reflected in flourishing roots, but that of *CP* is more prominently reflected in stomata regulation.

## Conclusion

In this study, both *MP* and *CP* were found to respond to drought through a fast reduction in canopy transpiration and canopy stomatal conductance, which was attributed to their typical isohydric behavior. *MP* and *CP* also behaved as water savers with relatively low daily water use intensity during the growing season. These findings provide basic information on the ability of pine trees to adapt to drought in semi-arid sandy land habitats. In comparison, *CP* showed less-conservative water use than did *MP* with higher canopy transpiration under favorable soil moisture condition. However, greater reductions in canopy transpiration and canopy stomatal conductance were found in *CP* than in *MP* under developing drought. *CP* is more sensitive to water availability, with significant inter-seasonal variability in canopy transpiration and a rapid decline in canopy stomatal conductance with increasing vapor pressure deficit than observed for *MP*. These results imply a more obvious isohydric behavior in *CP* than in *MP* through tighter stomatal regulation of water use in response to drought. From this point of view, as an introduced tree species, *MP* may increasingly face problems related to drought stress and thus has less adaptive capacity in this semi-arid sandy land. Our study highlights the similarities and differences in water use patterns of the two co-existing pine species, and provides suggestions for establishing a mixed shelterbelt forests that adapt to the local hydrological environment and flexibly respond to climate change.

## Data Availability Statement

The raw data supporting the conclusions of this article will be made available by the authors, without undue reservation.

## Author Contributions

HD provided the idea for this research and prepared the manuscript. XZ and HH helped to carry out the measurement. SC and ML collected the data in the study site. All authors contributed to the article and approved the submitted version.

## Conflict of Interest

The authors declare that the research was conducted in the absence of any commercial or financial relationships that could be construed as a potential conflict of interest.

## Footnotes

1 http://cran.r-project.org/web/packages/SPEI

2 https://dynamax.com/products/transpiration-sap-flow/tdp-sap-velocity-thermal-dissipation-probe

3 https://cran.r-project.org/web/packages/quantreg/

## References

[B1] Addington R. N. Mitchell R. J. Oren R. Donovan L. A. (2004). Stomatal sensitivity to vapor pressure deficit and its relationship to hydraulic conductance in *Pinus palustris*. *Tree Physiol.* 24 561–569. 10.1093/treephys/24.5.561 14996660

[B2] Allen C. D. Macalady A. K. Chenchouni H. Bachelet D. McDowell N. Vennetier M. (2010). A global overview of drought and heat-induced tree mortality reveals emerging climate change risks for forests. *For. Ecol. Manag.* 259 660–684. 10.1016/j.foreco.2009.09.001

[B3] Anderegg W. R. Konings A. G. Trugman A. T. Yu K. Bowling D. R. Gabbitas R. (2018). Hydraulic diversity of forests regulates ecosystem resilience during drought. *Nature* 561 538–541. 10.1038/s41586-018-0539-7 30232452

[B4] Bai J. Wang Z. Chen F. Li T. Han G. (2008). Water physiology of *Pinus edulis Engelm*., *Pinus tabulaeformis* and *Pinus sylvestris* var. mongolica under drought stress. *J. Northwest For. Univ.* 23 10–13.

[B5] Beguería S. Vicente Serrano S. M. Reig F. Latorre B. (2014). Standardized precipitation evapotranspiration index (SPEI) revisited: parameter fitting, evapotranspiration models, tools, datasets and drought monitoring. *Int. J. Climatol.* 34 3001–3023. 10.1002/joc.3887

[B6] Bernier P. Y. Bréda N. Granier A. Raulier F. Mathieu F. (2002). Validation of a canopy gas exchange model and derivation of a soil water modifier for transpiration for sugar maple (*Acer saccharum* Marsh.) using sap flow density measurements. *For. Ecol. Manag.* 163 185–196. 10.1016/s0378-1127(01)00578-3

[B7] Børja I. Svĕtlík J. Nadezhdin V. Čermák J. Rosner S. Nadezhdina N. (2016). Sap flux - a real time assessment of health status in Norway spruce. *Scand. J. For. Res.* 31 450–457. 10.1080/02827581.2015.1130851

[B8] Bovard B. D. Curtis P. S. Vogel C. S. Su H. B. Schmid H. P. (2005). Environmental controls on sap flow in a northern hardwood forest. *Tree Physiol.* 25 31–38. 10.1093/treephys/25.1.31 15519983

[B9] Bryan B. A. Gao L. Ye Y. Sun X. Connor J. D. Crossman N. D. (2018). China’s response to a national land-system sustainability emergency. *Nature* 559 193–204. 10.1038/s41586-018-0280-2 29995865

[B10] Buras A. Schunk C. Zeitrag C. Herrmann C. Kaiser L. Lemme H. (2018). Are Scots pine forest edges particularly prone to drought-induced mortality? *Environ. Res. Lett.* 13:025001. 10.1088/1748-9326/aaa0b4

[B11] Cai L. Li J. Bai X. Jin Y. Chen Z. (2020). Variations in the growth response of *Pinus tabulaeformis* to a warming climate at the northern limits of its natural range. *Trees* 34 707–719. 10.1007/s00468-019-01950-2

[B12] Campbell G. S. Norman J. M. (1998). *An Introduction to Environmental Biophysics.* New York, NY: Springer.

[B13] Chen C. Park T. Wang X. Piao S. Xu B. Chaturvedi R. K. (2019). China and India lead in greening of the world through land-use management. *Nat. Sustain.* 2 122–129. 10.1038/s41893-019-0220-7 30778399 PMC6376198

[B14] Chen H. Sun J. (2015). Changes in drought characteristics over China using the standardized precipitation evapotranspiration index. *J. Clim.* 28 5430–5447. 10.1175/jcli-d-14-00707.1

[B15] Chirino E. Bellot J. Sanchez J. R. (2011). Daily sap flow rate as an indicator of drought avoidance mechanisms in five Mediterranean perennial species in semi-arid southeastern Spain. *Trees* 25 593–606. 10.1007/s00468-010-0536-4

[B16] Choat B. Brodribb T. J. Brodersen C. R. Duursma R. A. López R. Medlyn B. E. (2018). Triggers of tree mortality under drought. *Nature* 558 531–539. 10.1038/s41586-018-0240-x 29950621

[B17] Cook B. I. Mankin J. S. Anchukaitis K. J. (2018). Climate change and drought: from past to future. *Curr. Clim. Change Rep.* 4 164–179. 10.1007/s40641-018-0093-2

[B18] Dang H. Lu P. Yang W. Han H. Zhang J. (2019a). Drought-induced reductions and limited recovery in the radial growth, transpiration, and canopy stomatal conductance of Mongolian Scots pine (*Pinus sylvestris* var. mongolica Litv): a five-year observation. *Forests* 10:1143. 10.3390/f10121143

[B19] Dang H. Zhang L. Yang W. Feng J. Han H. Chen Y. (2019b). Severe drought strongly reduces water use and its recovery ability of mature Mongolian Scots pine (*Pinus sylvestris* var. mongolica Litv.) in a semi-arid sandy environment of northern China. *J. Arid Land* 11 880–891. 10.1007/s40333-019-0029-2

[B20] Ding X. He Q. Zhang F. Liu Y. (2011). The photosynthetic characteristics of *Pinus Sylvestris* var. mongolica and *Pinus Tabuleaformis* in Mu Us sandland. *Res. Soil Water Conserv.* 18 215–219.

[B21] Dulamsuren C. Hauck M. Bader M. Oyungerel S. Osokhjargal D. Nyambayar S. (2009). The different strategies of *Pinus sylvestris* and *Larix sibirica* to deal with summer drought in a northern Mongolian forest–steppe ecotone suggest a future superiority of pine in a warming climate. *Can. J. For. Res.* 39 2520–2528. 10.1139/x09-156

[B22] Epron D. Osawa A. (2017). Fine roots: when anisotropy matters. *Tree Physiol.* 37 693–696. 10.1093/treephys/tpx063 28541584

[B23] Ewers B. E. Oren R. (2000). Analyses of assumptions and errors in the calculation of stomatal conductance from sap flux measurements. *Tree Physiol.* 20 579–589. 10.1093/treephys/20.9.579 12651422

[B24] Feng X. Ackerly D. D. Dawson T. E. Manzoni S. McLaughlin B. Skelton R. P. (2019). Beyond isohydricity: the role of environmental variability in determining plant drought responses. *Plant Cell Environ.* 42 1104–1111. 10.1111/pce.13486 30513545

[B25] Garcia-Forner N. Biel C. Save R. Martinez-Vilalta J. (2017). Isohydric species are not necessarily more carbon limited than anisohydric species during drought. *Tree Physiol.* 37 441–455. 10.1093/treephys/tpw109 27885172

[B26] Giuggiola A. Kuster T. M. Saha S. (2010). Drought-induced mortality of Scots pines at the southern limits of its distribution in Europe: causes and consequences. *Iforest* 3 95–97. 10.3832/ifor0542-003 17959540

[B27] Granier A. (1987). Evaluation of transpiration in a Douglas-fir stand by means of sap flow measurements. *Tree Physiol.* 3 309–320. 10.1093/treephys/3.4.309 14975915

[B28] Granier A. Breda N. Biron P. Villette S. (1999). A lumped water balance model to evaluate duration and intensity of drought constraints in forest stands. *Ecol. Model.* 116 269–283. 10.1016/s0304-3800(98)00205-1

[B29] Guada G. Camarero J. J. Sánchez-Salguero R. Cerrillo R. M. N. (2016). Limited growth recovery after drought-induced forest dieback in very defoliated trees of two pine species. *Front. Plant Sci.* 7:418. 10.3389/fpls.2016.00418 27066053 PMC4817349

[B30] Guo J. S. Ogle K. (2019). Antecedent soil water content and vapor pressure deficit interactively control water potential in *Larrea tridentata*. *New Phytol.* 221 218–232. 10.1111/nph.15374 30129140

[B31] Han H. Bai X. Xu G. Zhang B. You G. Liu S. (2013). Trunk sap flux density of *Pinus sylvestris* var. mongolica in Zhanggutai area. *J. Northeast For. Univ.* 41 27–31.

[B32] Heres A. M. Camarero J. J. Lopez B. C. Martinez-Vilalta J. (2014). Declining hydraulic performances and low carbon investments in tree rings predate Scots pine drought-induced mortality. *Trees Struct. Funct.* 28 1737–1750. 10.1007/s00468-014-1081-3

[B33] Hernandez-Santana V. Rodriguez-Dominguez C. M. Fernandez J. E. Diaz-Espejo A. (2016). Role of leaf hydraulic conductance in the regulation of stomatal conductance in almond and olive in response to water stress. *Tree Physiol.* 36 725–735. 10.1093/treephys/tpv146 26846979

[B34] Hochberg U. Rockwell F. E. Holbrook N. M. Cochard H. (2018). Iso/anisohydry: a plant–environment interaction rather than a simple hydraulic trait. *Trends Plant Sci.* 23 112–120. 10.1016/j.tplants.2017.11.002 29223922

[B35] Irvine J. Perks M. P. Grace J. (1998). The response of *Pinus sylvestris* to drought: stomatal control of transpiration and hydraulic conductance. *Tree Physiol.* 18 393–402. 10.1093/treephys/18.6.393 12651364

[B36] Jiang F. Cao C. Zeng D. (2002). *Degradation and Restoration of Ecosystems on Keerqin Sandy Land.* Beijing: Chinese Forestry Press.

[B37] Jiang P. Wang H. Meinzer F. C. Kou L. Dai X. Fu X. (2020). Linking reliance on deep soil water to resource economy strategies and abundance among coexisting understorey shrub species in subtropical pine plantations. *New Phytol.* 225 222–233. 10.1111/nph.16027 31247133

[B38] Jiao S. (2001). Report on the causes of the early decline of *Pinus slyvestris* var. mongolica shelterbelt and its preventative and control measures in Zhanggutai of Liaoning Province. *Sci. Silvae Sin.* 37 131–138.

[B39] Jones H. G. (1984). *Plants and Microclimate: A Quantitative Approach to Environmental Plant Physiology.* Cambridge: Cambridge University Press.

[B40] Klein T. (2015). Drought-induced tree mortality: from discrete observations to comprehensive research. *Tree Physiol.* 35 225–228. 10.1093/treephys/tpv029 25852087

[B41] Konôpka B. Yuste J. C. Janssens I. A. Ceulemans R. (2005). Comparison of fine root dynamics in Scots pine and pedunculate oak in sandy soil. *Plant Soil* 276 33–45. 10.1007/s11104-004-2976-3

[B42] Köstner B. Biron P. Siegwolf R. Granier A. (1996). Estimates of water vapor flux and canopy conductance of scots pine at the tree level utilizing different xylem sap flow methods. *Theor. Appl. Climatol.* 53 105–113. 10.1007/bf00866415

[B43] Kume T. Onozawa Y. Komatsu H. Tsuruta K. Shinohara Y. Umebayashi T. (2010). Stand-scale transpiration estimates in a Moso bamboo forest: (I) applicability of sap flux measurements. *For. Ecol. Manag.* 260 1287–1294. 10.1016/j.foreco.2010.07.012

[B44] Leo M. Oberhuber W. Schuster R. Grams T. E. E. Matyssek R. Wieser G. (2013). Evaluating the effect of plant water availability on inner alpine coniferous trees based on sap flow measurements. *Eur. J. For. Res.* 133 691–698. 10.1007/s10342-013-0697-y

[B45] Licata J. A. Gyenge J. E. Fernandez M. E. Schlichter T. A. Bond B. J. (2008). Increased water use by ponderosa pine plantations in northwestern Patagonia, Argentina compared with native forest vegetation. *For. Ecol. Manag.* 255 753–764. 10.1016/j.foreco.2007.09.061

[B46] Liu N. Bao G. Bao M. (2019). Response characteristics of Chinese pine (*Pinus tabulaeformis* carr.) radial growth to climate and drought variability reconstruction in western Liaoning, northeast China. *Forests* 10:752. 10.3390/f10090752

[B47] Llorens P. Poyatos R. Latron J. Delgado J. Oliveras I. Gallart F. (2010). A multi-year study of rainfall and soil water controls on Scots pine transpiration under Mediterranean mountain conditions. *Hydrol. Process.* 24 3053–3064. 10.1002/hyp.7720

[B48] Lu P. Urban L. Zhao P. (2004). Granier’s thermal dissipation probe (TDP) method for measuring sap flow in trees: theory and practice. *Acta Bot. Sin.* 46 631–646.

[B49] Ma L. Sun P. Ma L. (2001). Sapwood area calculation and water use scaling up from individual trees to stands of Chinese pine and black locust. *J. Beijing For. Univ.* 23:e077.

[B50] Macinnis-Ng C. Wyse S. Veale A. Schwendenmann L. Clearwater M. (2016). Sap flow of the southern conifer, *Agathis australis* during wet and dry summers. *Trees* 30 19–33. 10.1007/s00468-015-1164-9

[B51] Makkonen K. Helmisaari H. (2001). Fine root biomass and production in Scots pine stands in relation to stand age. *Tree Physiol.* 21 193–198. 10.1093/treephys/21.2-3.193 11303650

[B52] Martinez-Vilalta J. Pinol J. (2002). Drought-induced mortality and hydraulic architecture in pine populations of the NE Iberian Peninsula. *For. Ecol. Manag.* 161 247–256. 10.1016/s0378-1127(01)00495-9

[B53] McDowell N. Pockman W. T. Allen C. D. Breshears D. D. Cobb N. Kolb T. (2008). Mechanisms of plant survival and mortality during drought: why do some plants survive while others succumb to drought? *New Phytol.* 178 719–739. 10.1111/j.1469-8137.2008.02436.x 18422905

[B54] McDowell N. G. Ryan M. G. Zeppel M. J. Tissue D. T. (2013). Improving our knowledge of drought-induced forest mortality through experiments, observations, and modeling. *New Phytol.* 200 289–293. 10.1111/nph.12502 24050629

[B55] Meir P. Mencuccini M. Dewar R. C. (2015). Drought-related tree mortality: addressing the gaps in understanding and prediction. *New Phytol.* 207 28–33. 10.1111/nph.13382 25816852

[B56] Monteith J. Unsworth M. (1990). *Principles of Environmental Physics.* London: Edward Arnold.

[B57] Moore G. W. Bond B. J. Jones J. A. Meinzer F. C. (2010). Thermal-dissipation sap flow sensors may not yield consistent sap-flux estimates over multiple years. *Trees* 24 165–174. 10.1007/s00468-009-0390-4

[B58] Morán-López T. Poyatos R. Llorens P. Sabaté S. (2014). Effects of past growth trends and current water use strategies on Scots pine and pubescent oak drought sensitivity. *Eur. J. For. Res.* 133 369–382. 10.1007/s10342-013-0768-0

[B59] Nadezhdina N. (1999). Sap flow index as an indicator of plant water status. *Tree Physiol.* 19 885–891. 10.1093/treephys/19.13.885 10562406

[B60] Ogle K. Lucas R. W. Bentley L. P. Cable J. M. Barron Gafford G. A. Griffith A. (2012). Differential daytime and night-time stomatal behavior in plants from North American deserts. *New Phytol.* 194 464–476. 10.1111/j.1469-8137.2012.04068.x 22348404

[B61] Oren R. Sperry J. S. Katul G. G. Pataki D. E. Ewers B. E. Phillips N. (1999). Survey and synthesis of intra- and interspecific variation in stomatal sensitivity to vapour pressure deficit. *Plant Cell Environ.* 22 1515–1526. 10.1046/j.1365-3040.1999.00513.x

[B62] Payn T. Carnus J. Freer-Smith P. Kimberley M. Kollert W. Liu S. (2015). Changes in planted forests and future global implications. *For. Ecol. Manag*. 352 57–67. 10.1016/j.foreco.2015.06.021

[B63] Peters M. P. Iverson L. R. Matthews S. N. (2015). Long-term droughtiness and drought tolerance of eastern US forests over five decades. *For. Ecol. Manag.* 345 56–64. 10.1016/j.foreco.2015.02.022

[B64] Poyatos R. Aguadé D. Galiano L. Mencuccini M. Martínezvilalta J. (2013). Drought-induced defoliation and long periods of near-zero gas exchange play a key role in accentuating metabolic decline of Scots pine. *New Phytol.* 200 388–401. 10.1111/nph.12278 23594415

[B65] Poyatos R. Llorens P. Gallart F. (2005). Transpiration of montane *Pinus sylvestris* L. and *Quercus pubescens* Willd. Forest stands measured with sap flow sensors in NE Spain. *Hydrol. Earth Syst. Sci.* 9 493–505. 10.5194/hess-9-493-2005

[B66] Poyatos R. Llorens P. Pinol J. Rubio C. (2008). Response of Scots pine (*Pinus sylvestris* L.) and pubescent oak (*Quercus pubescens* Willd.) to soil and atmospheric water deficits under Mediterranean mountain climate. *Ann. For. Sci.* 65 306–318. 10.1051/forest:2008003

[B67] Quan C. Xing X. Li Z. He Z. (2004). Comparative study on the introduction of *Pinus sylvestris* var.mongolica and *Pinus tabulaeformis* in Ejin Horo Banner, Inner Mongolia. *J. Beijing For. Univ.* 26 63–67.

[B68] Song L. Zhu J. Li M. Zhang J. Zheng X. Wang K. (2018). Canopy transpiration of *Pinus sylvestris* var. *mongolica* in a sparse wood grassland in the semiarid sandy region of Northeast China. *Agric. For. Meteorol*. 250–251 192–201. 10.1016/j.agrformet.2017.12.260

[B69] Song L. Zhu J. Yan Q. Li M. Yu G. (2015). Comparison of intrinsic water use efficiency between different aged *Pinus sylvestris* var. *mongolica* wide windbreaks in semiarid sandy land of northern China. *Agrofor. Syst*. 89 477–489. 10.1007/s10457-014-9784-4

[B70] Song L. Zhu J. Zheng X. Wang K. Lü L. Zhang X. (2020). Transpiration and canopy conductance dynamics of *Pinus sylvestris* var. mongolica in its natural range and in an introduced region in the sandy plains of Northern China. *Agric. For. Meteorol.* 281:107830. 10.1016/j.agrformet.2019.107830

[B71] Song L. N. Zhu J. J. Li M. C. Yu Z. Y. (2014). Water utilization of *Pinus sylvestris* var. mongolica in a sparse wood grassland in the semiarid sandy region of Northeast China. *Trees* 28 971–982. 10.1007/s00468-014-1010-5

[B72] Stocker T. F. Qin D. Plattner G. Tignor M. Allen S. K. (2013). *Climate Change 2013. The Physical Science Basis. Working Group? Contribution to the Fifth Assessment Report of the Intergovernmental Panel on Climate Change.* London: Cambridge University Press.

[B73] Sun J. Liu Y. (2014). Responses of tree-ring growth and crop yield to drought indices in the Shanxi province, North China. *Int. J. Biometeorol.* 58 1521–1530. 10.1007/s00484-013-0757-5 24162181

[B74] Tang Y. Wu X. Chen Y. (2018). Sap flow characteristics and physiological adjustments of two dominant tree species in pure and mixed plantations in the semi-arid Loess Plateau of China. *J. Arid Land* 10 833–849. 10.1007/s40333-018-0027-9

[B75] Tardieu F. Simonneau T. (1998). Variability among species of stomatal control under fluctuating soil water status and evaporative demand: modelling isohydric and anisohydric behaviours. *J. Exp. Bot.* 49 419–432. 10.1093/jexbot/49.suppl_1.419

[B76] Tatarinov F. Rotenberg E. Maseyk K. Ogée J. Klein T. Yakir D. (2016). Resilience to seasonal heat wave episodes in a Mediterranean pine forest. *New Phytol*. 210 485–496. 10.1111/nph.13791 27000955

[B77] Urban J. Rubtsov A. V. Urban A. V. Shashkin A. V. Benkova V. E. (2019). Canopy transpiration of a *Larix sibirica* and *Pinus sylvestris* forest in Central Siberia. *Agric. For. Meteorol.* 271 64–72. 10.1016/j.agrformet.2019.02.038

[B78] Vanguelova E. I. Kennedy F. (2007). Morphology, biomass and nutrient status of fine roots of Scots pine (*Pinus sylvestris*) as influenced by seasonal fluctuations in soil moisture and soil solution chemistry. *Plant Soil* 270 233–247. 10.1007/s11104-004-1523-6

[B79] Verbeeck H. Steppe K. Nadezhdina N. Beek M. O. Meiresonne L. Lemeur R. (2007). Stored water use and transpiration in Scots pine: a modeling analysis with ANAFORE. *Tree Physiol.* 27 1671–1685. 10.1093/treephys/27.12.1671 17938099

[B80] Vicente-Serrano S. M. Beguería S. López-Moreno J. I. (2010). A multiscalar drought index sensitive to global warming: the standardized precipitation evapotranspiration index. *J. Clim.* 23 1696–1718. 10.1175/2009jcli2909.1

[B81] Wang K. Guo J. Wang D. Liu S. Ni P. (2015). Resonses of roots and needles of *Pinus tabulaeformis* and *Pinus sylvestnis* var. mongolica to spring drought stress. *Chin. J. Ecol.* 34 3132–3138.

[B82] Wang X. M. Zhang C. X. Hasi E. Dong Z. B. (2010). Has the Three Norths Forest Shelterbelt Program solved the desertification and dust storm problems in arid and semiarid China? *J. Arid Environ.* 74 13–22. 10.1016/j.jaridenv.2009.08.001

[B83] Weber P. Bugmann H. Rigling A. (2007). Radial growth responses to drought of *Pinus sylvestris* and *Quercus pubescens* in an inner-Alpine dry valley. *J. Veg. Sci.* 18 777–792. 10.1111/j.1654-1103.2007.tb02594.x

[B84] Wen J. Chen Y. Tang Y. Wu X. Xie Y. Cui G. Y. (2017). Characteristics and affecting factors of sap flow density of *Pinus tabuliformis* and *Hippophae rhamnoides* in growing season in the hilly region of the Loess Plateau, China. *Chin. J. Appl. Ecol.* 28 763–771. 10.13287/j.1001-9332.201703.034 29741001

[B85] Wu X. Tang Y. Chen Y. Wen J. Xie Y. Lu S. (2018). Sap flow characteristics and responses to summer rainfall for *Pinus tabulaeformis* and *Hippophae rhamnoides* in the Loess hilly region of China. *Ecol. Evol.* 8 617–630. 10.1002/ece3.3639 29321898 PMC5756875

[B86] Xu J. (2011). China’s new forests aren’t as green as they seem. *Nature* 477:371. 10.1038/477371a 21938029

[B87] Xue W. (2003). Research on ecological characteristics of root system of main planting tree species in the Weibei areas. [Dissertation/Master’s thesis]. [Yangling]: *Northwest Sci. Tech. Univ. Agric. For.*

[B88] Yan C. Wang B. Zhang Y. Zhang X. Takeuchi S. Qiu G. Y. (2018). Responses of sap flow of deciduous and conifer trees to soil drying in a subalpine forest. *Forests* 9:32. 10.3390/f9010032

[B89] Zastrow M. (2019). China’s tree-planting drive could falter in a warming world. *Nature* 573 474–476. 10.1038/d41586-019-02789-w 31551554

[B90] Zhao X. Li W. (1963). *Pinus Sylvestris* var. mongolica. Beijing: China Agriculture Press.

[B91] Zheng X. Zhu J. J. Yan Q. L. Song L. N. (2012). Effects of land use changes on the groundwater table and the decline of *Pinus sylvestris* var. mongolica plantations in southern Horqin Sandy Land, Northeast China. *Agric. Water Manag.* 109 94–106. 10.1016/j.agwat.2012.02.010

[B92] Zhou Z. Shangguan Z. (2007). Vertical distribution of fine roots in relation to soil factors in *Pinus tabulaeformis* Carr. Forest of the Loess Plateau of China. *Plant Soil* 291 119–129. 10.1007/s11104-006-9179-z

[B93] Zhu J. Zeng D. Kang H. Wu X. Fan Z. (2005). *Decline of Pinus sylvestris* var. mongolica plantations on Sandy Land. Beijing: China Forestry Publishing House.

[B94] Zhu J. Zheng X. (2019). The propects of development of the Three-North Afforestation Program (TNAP): on the basis of the results of the 40-year construction general assessment of the TNAP. *Chin. J. Ecol.* 38 1600–1610.

